# Specific Nuclear Localizing Sequence Directs Two Myosin Isoforms to the Cell Nucleus in Calmodulin-Sensitive Manner

**DOI:** 10.1371/journal.pone.0030529

**Published:** 2012-01-25

**Authors:** Rastislav Dzijak, Sukriye Yildirim, Michal Kahle, Petr Novák, Jarmila Hnilicová, Tomáš Venit, Pavel Hozák

**Affiliations:** 1 Department of Biology of the Cell Nucleus, Institute of Molecular Genetics of the ASCR, v.v.i., Prague, Czech Republic; 2 Laboratory of Molecular Structure Characterization, Institute of Microbiology of the ASCR, v.v.i., Prague, Czech Republic; Brunel University, United Kingdom

## Abstract

**Background:**

Nuclear myosin I (NM1) was the first molecular motor identified in the cell nucleus. Together with nuclear actin, they participate in crucial nuclear events such as transcription, chromatin movements, and chromatin remodeling. NM1 is an isoform of myosin 1c (Myo1c) that was identified earlier and is known to act in the cytoplasm. NM1 differs from the “cytoplasmic” myosin 1c only by additional 16 amino acids at the N-terminus of the molecule. This amino acid stretch was therefore suggested to direct NM1 into the nucleus.

**Methodology/Principal Findings:**

We investigated the mechanism of nuclear import of NM1 in detail. Using over-expressed GFP chimeras encoding for truncated NM1 mutants, we identified a specific sequence that is necessary for its import to the nucleus. This novel nuclear localization sequence is placed within calmodulin-binding motif of NM1, thus it is present also in the Myo1c. We confirmed the presence of both isoforms in the nucleus by transfection of tagged NM1 and Myo1c constructs into cultured cells, and also by showing the presence of the endogenous Myo1c in purified nuclei of cells derived from knock-out mice lacking NM1. Using pull-down and co-immunoprecipitation assays we identified importin beta, importin 5 and importin 7 as nuclear transport receptors that bind NM1. Since the NLS sequence of NM1 lies within the region that also binds calmodulin we tested the influence of calmodulin on the localization of NM1. The presence of elevated levels of calmodulin interfered with nuclear localization of tagged NM1.

**Conclusions/Significance:**

We have shown that the novel specific NLS brings to the cell nucleus not only the “nuclear” isoform of myosin I (NM1 protein) but also its “cytoplasmic” isoform (Myo1c protein). This opens a new field for exploring functions of this molecular motor in nuclear processes, and for exploring the signals between cytoplasm and the nucleus.

## Introduction

Nuclear myosin I (NM1) was the first unconventional myosin motor detected in the cell nucleus [1]. NM1 is an isoform of earlier identified cytoplasmic myosin Ic (Myo1c) containing additional 16 amino acids at the N-terminus. The mRNA of NM1 is differently spliced yielding 5′ introduction of exon containing alternative start of translation [2]. Importantly, the ubiquitous expression and nuclear localization of NM1 in mouse organs along with high degree of conservation of the N-terminal sequence across species has been confirmed [3], [4].

This corresponds to its important functions. In the nucleus, there is ample evidence for functional involvement of NM1 in transcription by RNA polymerase I and II (Pol I and Pol II). NM1 co-localizes with both polymerases at the sites of transcription [2], [5] and physically associates with both Pol I and Pol II complexes [6], [7]. In-vivo rate of transcription is negatively affected by NM1 overexpression, and inhibited by NM1 knock-down and nuclear microinjections of anti-NM1 antibodies [7]. In an in-vitro transcription system, anti-NM1 antibodies inhibit transcription by both polymerases in a dose-dependent manner, whereas adding purified NM1 increases transcription [2], [6], [8]. Transcription initiation assays have revealed that NM1 exerts its function in early steps of Pol I and II transcription after the formation of pre-initiation complexes [6], [7]. Indeed, NM1 interacts with Pol I transcription factor TIF-IA, which is present only in initiation-competent fraction of Pol I complexes [9], and actin that is associated with RNA polymerase I independently of active transcription [7]. According to Grummt [10], the binding of NM1 to Pol I via actin may help to initiate transcription by recruiting TIF-IA to pre-initiation complex. This model is further supported by the fact that functional motor domain is needed for interaction of NM1 and Pol I [11]. In addition to transcription initiation, NM1 is also involved in Pol I transcription elongation since it associates with the chromatin remodeling complex WSTF-SNF2h and might therefore recruit this complex to the actively transcribing genes [12].

Interestingly, nascent ribosomal particles seem to be accompanied by NM1 during transport from nucleolus toward nuclear pores [13] and blocking of NM1 or actin by antibodies results in nuclear retention of small ribosomal subunits [14], [15].

A role of acto-myosin motor in repositioning of chromosomes is emerging [16], [17]. In pioneering work, Chuang and co-workers [18] showed that labeled artificial gene loci move, upon activation, toward the center of nucleus and that overexpression of mutated NM1 that lacks motor activity inhibits this effect. However, the exact mechanism behind these translocation phenomena is not clear.

Using specific antibodies generated against its N-terminal epitope, NM1 can be detected predominantly in the nucleus, nucleolus and at the plasma membrane of interphase cells [1], [5], [19]. NM1 is a short-tailed class I myosin that binds directly to actin via its head domain and the headgroups of acidic phospholipids via putative PH domain within positively charged tail [20]. Neck domain, located between head and tail, contains three IQ motifs that bind calmodulin [1]. To date, there are no data about biochemical characteristics of this protein. Because NM1 is almost identical to Myo1c, one can expect that its basic function is to maintain tensions as proposed for Myo1c [21] however, the exact function of the N-terminal extension in NM1 molecule that makes the only known difference form Myo1c is uncertain. The observation that NM1 is localized mainly in the nucleus and Myo1c at the plasma membrane has led to the opinion that the N-terminus could function as a nuclear targeting or nuclear sequestering sequence [2].

In this paper we identify the domains that direct the nuclear translocation of NM1 and decipher the mechanism of intracellular trafficking of NM1. We demonstrate that the N-terminal extension of NM1does not act as a nuclear localization sequence (NLS); instead, we identified a novel NLS within the the neck region of NM1 as crucial for nuclear import. In search for the possible import receptors of NM1 we found importin 5, importin-β1, importin 7 and Heat shock protein 90 (HSP90) to associate with truncated constructs as well as with the endogenous NM1. Since the identified NLS sequence is also present in the Myo1c protein we also investigated the localization of Myo1c. Using various experiments including the NM1 knockout mice derived cells we discovered the “cytoplasmic” Myo1c was also present in nuclei. This adds the traditional “cytoplasmic” Myo1c to the few molecular motors of the nucleus with potentially important functions in nuclear metabolism.

## Results

### NM1 is transported to the nucleus after mitosis

**Figure 1:**
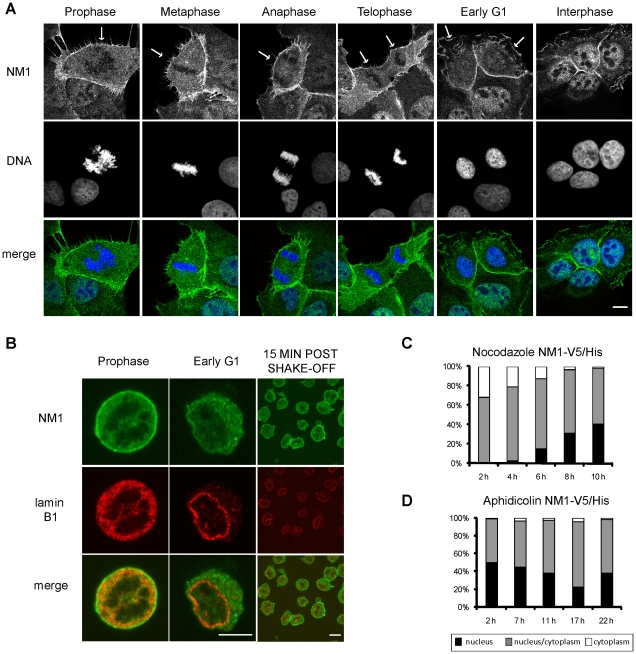
Localization NM1 during mitosis. (**A**) Unsynchronized U2OS cells were fixed and labeled with antibody to NM1. Localization of NM1 is shown at various stages of mitosis. DNA was visualized by DAPI. (**B**) Mitotic NIH 3T3 cell were seeded onto the poly-L-lysine coated coverslips, fixed and labeled with antibodies to NM1 and Lamin B1. Cells fixed immediately after seeding (**Prophase**) and 15 min after seeding (**Early G1, 15 min post shake-off**). Nuclear lamina was reconstituted in Early G1 as visualized by Lamin B1 labeling. All immunofluorescence pictures were obtained using confocal microscope, single confocal sections are shown. Scale bar: 10 µm. U2OS cells were transiently transfected with NM1-V5/His. 24 hours after transfection cells were treated with nocodazole (**C**) or aphidicolin (**D**), to stall the cells either in G2/M or in G1/S phase of cell cycle. After the release from the block cells were cultivated for another 24 hours. Samples were taken in indicated time points. Cells were labeled with antibody to V5 tag, patterns counted and divided into three groups according to the localization of fluorescent proteins. More than 100 cells were counted in each time point, experiment was repeated twice with similar result.

To study the dynamics of NM1 compartmentalization during cell cycle we followed the localization pattern of the endogenous NM1 during and after mitosis. Immunofluorescent labeling of NM1 in unsynchronized U2OS (Fig. 1A) and in NIH 3T3 (Fig. 1B) cells synchronized by mitotic shake off has shown that NM1 did not stay bound to chromatin during the mitosis and that its majority was released into the cytoplasm after the nuclear envelope breakdown in prophase (Fig. 1B). Soon after the reconstitution of nuclear envelope in early G1, most of NM1 was in the cytoplasm as shown in Fig. 1A and 1B (Early G1). In unsynchronized population of cells, this pattern was very rarely observed, and the vast majority of cells had clearly nuclear staining of NM1 (Fig. 1A, Interphase). This demonstrates that nuclear import of endogenous NM1 is accomplished in G1 phase.

To begin identifying import signals in NM1, we first tested the localization of full length NM1 constructs fused to different tags. Untagged overexpressed mouse and human NM1 localized predominantly in the nucleus in 80% of cells, and V5/His-tagged NM1 was predominantly nuclear in 50%, whereas EGFP-tagged or FLAG-tagged NM1 was predominantly nuclear in less than 20% of cells (data not shown). Further studies used the V5/His-tag because it interfered with nuclear import the least.

To visualize the timing of V5/His-tagged NM1 (NM1-V5/His) transport into the nucleus after mitosis, we transfected U2OS cells and the next day added either nocodazole (depolymerizes microtubules) or aphidicolin (DNA polymerase inhibitor) for 16 hours to accumulate cells in M-phase or S-phase respectively, then washed out the inhibitor and used indirect immunofluorescence to localize NM1-V5/His at different times after release from the block (Fig. 1C,D). Nocodazole-treated (metaphase-enriched) cells continued with mitosis after washout. The lowest nuclear levels of tagged NM1 were seen at 2 and 4 hours after release, but increased gradually at 6–10 hours after release (Fig. 1C). After release from aphidicolin, cells maintained high nuclear levels of NM1-V5/His for ∼11 hours, consistent with the expected time needed to complete S-phase and enter G2 (Fig. 1D). The lowest levels of nuclear NM1-V5/His were detected 17 hours after release from aphidicolin (Fig. 1D), when many cells were in mitosis or early G1. Together these results suggested both endogenous and tagged NM1 are released from the nucleus during mitosis. Endogenous NM1 is transported into renewed nuclei shortly after the nuclear envelope reconstitution in the early G1, while nuclear import of the ectopically expressed NM1 with a tag is slower.

### First two IQ domains are needed for nuclear transport of NM1

**Figure 2:**
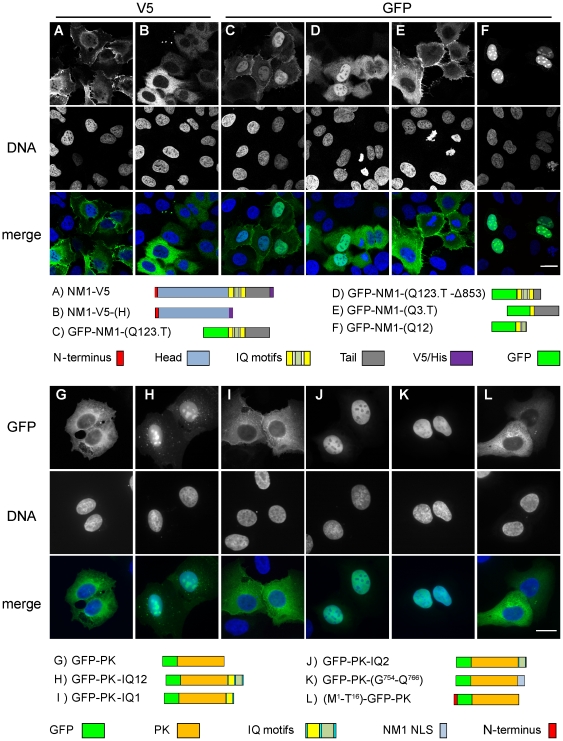
Neck domain of NM1 contains the NLS. U2OS cell transfected with a panel of truncation constructs of full length NM1 (**A–F**) and IQ domains fused to GFP-PK (**G–L**). Cells were fixed 48 hours post transfection. Below the pictures are schematic representations of the truncations affecting various NM1 domains as well as the GFP-PK phusions. Pictures (**A–F**) were acquired using confocal microscope, single confocal planes are shown. Pictures (**G–L**) were photographed using wide-field fluorescent microscope. Scale bar: 10 µm.

Because the N-terminal part of NM1 was suggested to be crucial for nuclear localization [2], we prepared various deletion and truncation mutants of the NM1 in fusion with V5/His at its C-terminus. We compared their localization in U2OS cells with the full length NM1-V5 which was detected in the cytoplasm and faintly in the nucleus (Fig. 2A, anti-V5). Surprisingly, the deletion of the neck and the tail domain led to the cytoplasmic retention of the mutant (Fig. 2B). This suggested that the NLS sequence is located within the neck or in the tail domains. After deletion of the head domain, we observed enhanced nuclear signal with short C-terminal V5/His (not shown) as well as with the bulky N-terminal EGFP tag (Fig. 2C). This suggested that the EGFP-fused myosin neck-tail fragment is imported efficiently. Further deletion of half of the tail disrupted the plasma membrane association of the protein but not its nuclear translocation (Fig. 2D). The tail together with the third IQ domain of the neck stayed out of the nucleus and associated with plasma membrane (Fig. 2E) while the construct with first two IQ domains was located exclusively to the nucleus and nucleoli (Fig. 2F). This localized a putative nuclear localizing sequence within the first two IQ domains of NM1 neck residues 712–770.

### The second IQ domain contains a novel NLS sequence

**Figure 3:**
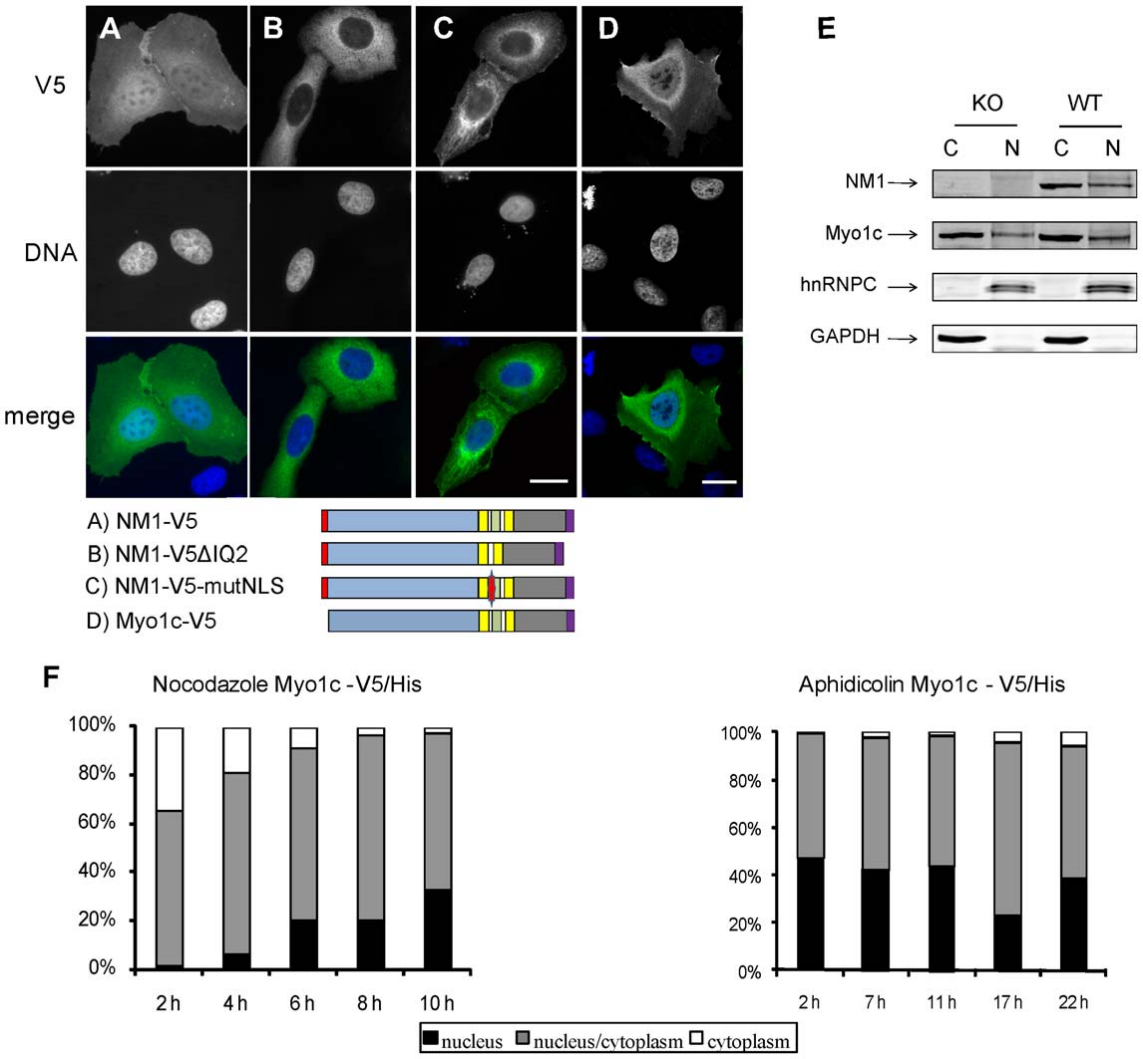
Mutation of basic residues in the neck of NM1/Myo1c abolishes its nuclear import. U2OS cells were transfected with full length NM1-V5/His (**A**), NM1-V5/His lacking the second IQ motif (**B**), and NM1-V5/His with point mutation of basic amino acids within the NLS into alanines (**C**). Below the pictures are schematic representations of constructs used. Color coding is the same as in **Fig. 2**. Cells were fixed 48 hours post transfection and labeled with anti-V5 antibody, pictures were obtained using wide-field microscope, scale bar: 10 µm (**D**) U2OS cells transiently transfected with Myo1c-V5/His show nuclear localization of the protein Picture is a single confocal plane, obtained by confocal microscope. Scale bar: 10 µm. (**E**) Nuclear and cytosolic extracts were prepared from liver of either wild type (WT) or NM1 knock-out (KO) mice. Equal amount of protein was resolved using SDS-PAGE and electro-transferred to nitrocellulose. Membrane was probed with anti-NM1, anti-Myo1c, anti hnRNP C1/C2 and GAPDH antibody. Signal was detected using LI-COR Odyssey infrared imaging system. (**F**) U2OS cells were transiently transfected with Myo1c-V5/His. 24 hours after transfection cells were treated with nocodazole or aphidicolin to stall the cells either in G2/M or in G1/S phase of cell cycle. After the release from the block cells were cultivated for another 24 hours. Samples were taken in indicated timepoints. Cells were labeled with antibody to V5 tag, patterns counted and divided into three groups according to the localization of fluorescent proteins. More than 100 cells were counted in each timepoint, expreriment was repeated twice with similar result.

To pinpoint the exact part of the neck needed for nuclear translocation, we prepared a set of fusion constructs containing GFP and the cytosolic pyruvate kinase (PK) enzyme [22]. We used PK to enlarge the proteins so that they would not diffuse passively to the nucleus as GFP alone would [23]. The 87 kDa GFP-PK fusion construct was located solely to the cytoplasm (Fig. 2G). When the sequence of the first two IQ domains was added to GFP-PK strong nuclear and nucleolar signal was observed (Fig. 2H). Next, we examined the capability of each IQ domain to drive the nuclear transport (Fig. 2I, J). Nuclear accumulation was specifically driven by the second IQ motif (Fig. 2J), not the first IQ motif (Fig. 2I). The IQ2 motif and its c-terminal flanking sequence contains two clusters of basic amino acids. Next, we preserved only the basic amino acid clusters with the intermitting non-polar amino acids, resulting in 13 amino acid peptide, ^754^GRRKAAKRKWAAQ^766^. This sequence was sufficient for nuclear translocation (Fig. 2K). On the other hand, the N-terminal 16 amino acids from NM1, fused to the N-terminus of the GFP-PK construct, did not localize to the nucleus at all (Fig. 2L). To rule out the possibility that it serves as a nuclear retention signal, we fused the N-terminal sequence to EGFP that diffuses freely into nucleus. We did not observe nuclear enrichment of the signal that would be caused by an interaction of the protein inside the nucleus (not shown). In contrast to the full length NM1 (Fig. 3A), a C-terminally fused NM1 construct lacking the residues 739–762 accumulated in the cytoplasm of U2OS cells (Fig. 3B), supporting an important role for the second IQ. No single K/R-to-A substitution in residues 754–766 was able to disrupt the NLS activity (data not shown). However mutating all 6 basic residues to Ala completely abolished nuclear import (Fig. 3C; NM1-V5-mutNLS). NLS Database [24] and literature searches revealed no known NLS homologous to that of NM1. We therefore conclude that NM1 and cytoplasmic Myo1c share a novel type of NLS.

### Myo1c is able to translocate into the nucleus

N-terminus of NM1 alone did not possess a nuclear localization potential and the NLS was located in region shared by both the NM1 and Myo1c. We therefore inspected the localization of Myo1c under overexpressed condition. V5/His tagged Myo1c was localized to the nuclei of transfected U2OS cells (Fig. 3D). The nuclear localization of Myo1c-V5/His in nocodazole- or aphidicolin-treated cells was also cell cycle dependent, with profiles (Fig. 3F) similar to that of cells that overexpressed NM1 (Fig. 1F).

**Figure 4:**
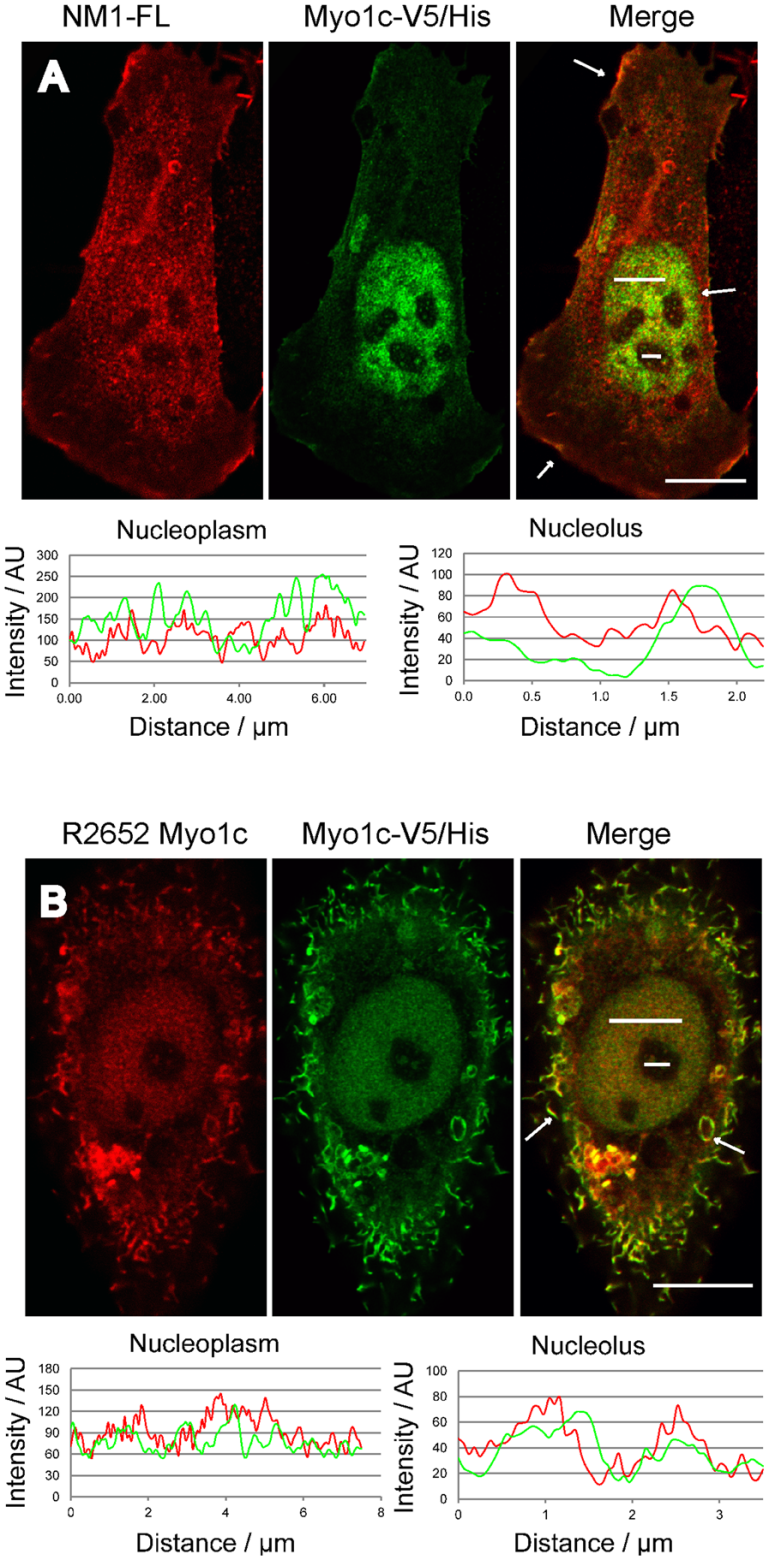
Co-localization of overexpressed NM1 and Myo1c. U2OS cells were co-transfected with FLAG-tagged NM1 (**NM1-FL**) and V5/His tagged Myo1c (**Myo1c-V5/His**). Cell showing nuclear localization of NM1 and Myo1c was photographed using confocal microscope (**A**). U2OS were transfected with Myo1c-V5/His. 48 h post transfection cells were fixed, and labeled with polyclonal antibody (R2652) directed toward the tail region of NM1/Myo1c and with monoclonal antibody against V5 (**B**). Intensity profiles along the regions of interest in the nucleus and nucleolus are shown under the pictures. White arrows are pointing to regions at the plasma membrane where both proteins are enriched. Scale Bar: 10 µm.

To test the influence of the N-terminal 16 amino acids on NM1 functions we prepared knock-out mice lacking the exon-1 that contains the NM1 start codon (Venit et al, in preparation). Resulting mRNA contains only the downstream start of translation which gives rise to Myo1c protein. We used purified liver nuclei from NM1 knock-out mice to confirm that the N-terminus is not required for nuclear transport of endogenous NM1. Fig. 3E shows the presence of Myo1c in the purified liver nuclei from NM1 knock-out mice visualized by antibody to the C-terminus of NM1/Myo1c. Weak signal of GAPDH indicates negligible contamination of nuclei with cytosol, while signal of hnRNP C1/C2 shows marked enrichment of nuclear proteins compared to cytosolic liver extract. This shows that both NM1 and Myo1c have the ability to enter the nucleus. Further, we have observed the co-localization of NM1 and myo1c in a single cell. However, we were not able to co-localize NM1 and myo1c in nuclei of untransfected cells using the polyclonal antibody directed to the tail region of NM1/Myo1c (R2652) [25] since it failed to label endogenous epitopes within nuclear environment. Therefore, U2OS cells were cotransfected with FLAG tagged NM1 and V5/His tagged Myo1c. Fig. 4A shows a cell expressing both proteins. Myo1c and NM1 co-localized at the plasma membrane (arrows) and in the nucleus. Interestingly, R2652 antibody revealed the signal in nucleus and nucleolus when myo1c was overexpressed Fig. 4B.

### Importins bind the NM1 neck region

**Figure 5:**
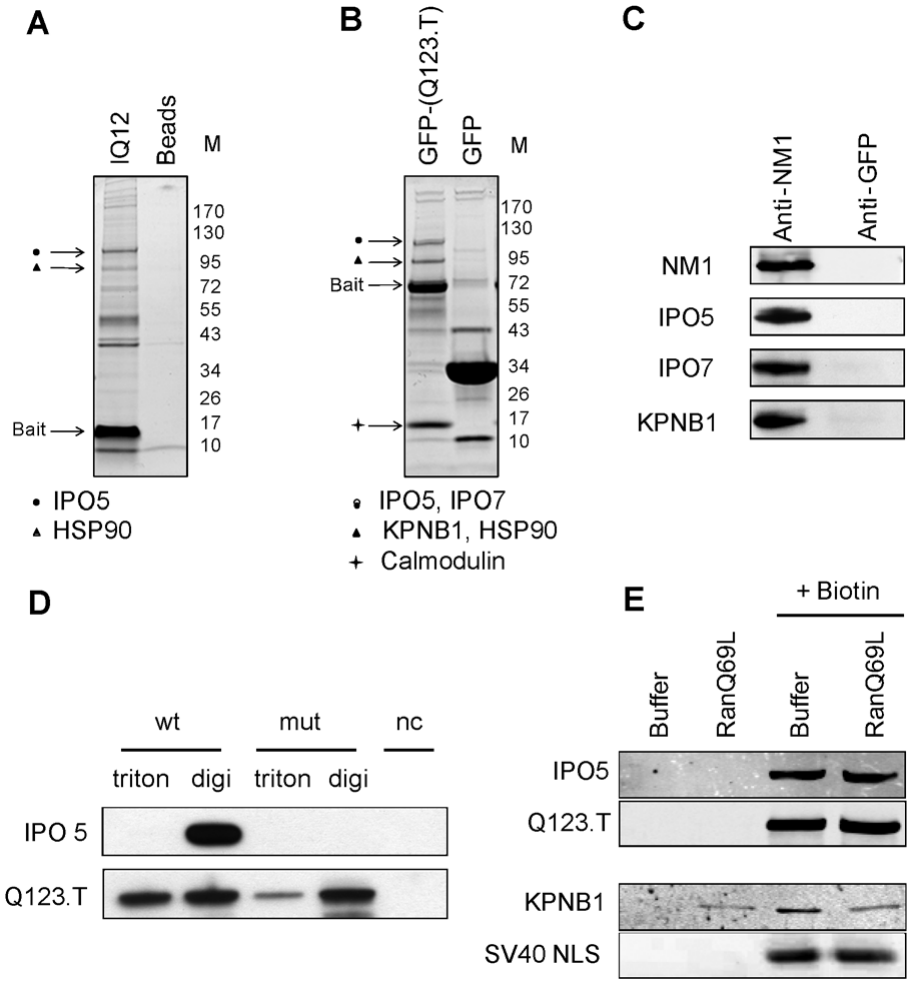
Identification of NM1 interacting proteins in the cytosol. Digitonin extract from suspension HeLa cells was incubated with recombinant Str-IQ12-His peptide containing N-terminal OneStrep tag (IQ12) and Streptactin beads as a control for background binding. Bound proteins were resolved on 4–20% SDS-PAGE gel and stained with SimplyBlue. Mass spectrometric analysis of the protein bands that co-purified with bait (arrows) identified importin 5 and heat shock protein 90 beta (HSP90) (**A**). SimplyBlue stained 4–20% SDS-PAGE gel with proteins that interacted with Str-GFP-NM1-(Q123.T) and Str-GFP as a control in digitonin extract of HEK293T cells. The arrows show positions of bands that contained proteins identified using mass spectrometry as importin 5, importin 7, importin-β1, HSP90 beta and calmodulin (**B**). Proteins that co-immunoprecipitate with antibody to endogenous NM1 from HeLa extracts were resolved using SDS-PAGE and tranferred onto nitrocelulose membrane. Membrane was probed with with anti-NM1, anti-importin 5 (IPO5), anti-importin 7 (IPO7), anti-importin-β1 (KPNB1). Rabbit polyclonal antibody against GFP was used as a control for backgroung binding (**C**). N-terminally Strep tagged GFP-NM1-(Q123.T) ^NLSwt^ (wt), GFP-NM1-(Q123.T) ^NLSmut^ (mut) and GFP as negative control (nc) were expressed in HEK293T cells. Cells were extracted with buffer containing digitonin (digi) to obtain soluble cytosol; pellet was re-extracted with the same buffer containing 1% Triton X-100 (triton). Bound proteins were resolved on SDS-PAGE, transferred to nitrocelulose. Membrane was incubated with antibody to importin 5 ans GFP (**D**). Beads containing Str-GFP-NM1-(Q123.T) and Str-GFP-SV40 NLS and associated proteins were eluted first with buffer containing GTP-loaded RanQ69L or buffer alone and then with biotin containig buffer that liberated Strep-tagged bait proteins from the column. Proteins eluted from the beads were resolved on SDS-PAGE and transferred to nitrocelulose membrane. GFP, importin 5 and importin-β1 signals were detected using specific antibodies (**E**). Signal from secondary antibodies was detected using LI-COR Odyssey infrared imaging system.

The transport of nuclear proteins through nuclear pores is often facilitated by importins that recognize their NLS in cytoplasm [24]. To discriminate between cytoplasmic and nucleus/plasma membrane-associated myosin, cells were extracted with buffers containing digitonin that is known to extract cytosolic myosin 1c [26]. We sought to identify the transport receptors that bind NM1/Myo1c NLS. Using pull down assay with recombinant IQ12 as the bait we identified importin 5 (IPO5) and Heat shock protein 90 beta (HSP90) as the proteins that associate with IQ12 in the cytoplasm of HeLa cells (Fig. 5A). To verify the obtained result by another method, we looked for interacting partners of GFP-NM1-(Q123.T) in HEK-293T cells. Mass spectrometry analysis of bands that co-purify specifically with GFP-NM1-(Q123.T) but not with the control Str-GFP contruct, revealed importin 5, importin 7 (IPO7), importin-β1 (KPNB1) and HSP90 beta (Fig. 5B). Additional bands that were present on the gel were not identified. To verify that the importin 5, importin 7 and importin-β1, which were found to bind the truncated constructs, recognize also the endogenous protein, we performed co-immunoprecipitation with a polyclonal antibody directed to N-terminus of NM1. Since most of endogenous NM1 molecules potentially accessible to importins are located in cytosol of the G1 cells (Fig. 1A), we synchronized the HeLa cells with nocodazole and harvested them 3 hours after nocodazole wash-out. As shown by western blot (Fig. 5C), endogenous NM1 specifically binds to importin 5 (IPO5), importin 7 (IPO7), and importin-β1 (KPNB1) in digitonin extracts of the G1 cells.

Next, to confirm that importin 5 binds specifically to NM1 NLS via the interaction with positively charged amino acids, we compared the proteins that co-purify with headless NM1 with wild type NLS (GFP-NM1-(Q123.T) ^NLSwt^) and headless NM1 with all basic residues in the NLS mutated to alanines (GFP-NM1-(Q123.T) ^NLSmut^) from electroporated HEK293T cells. Fig. 5D shows that importin 5 interacts only with GFP-NM1-(Q123.T) ^NLSwt^ and that this interaction occurs in digitonin extract in contrast to triton X-100 that liberates the plasma membrane bound myosin [26]. Taken together, the aforementioned data show that the importin 5, importin 7 and importin-β1 bind the newly identified NLS.

### NM1 nuclear import does not follow the canonical nuclear import pathway

The direction of canonical nuclear import pathway is controlled by the small GTPase Ran. High levels of GTP-loaded Ran in the nucleoplasm cause the dissociation of importin-cargo complex upon translocation through the nuclear pore complex [27]. We probed the stability of the NM1-importin complexes in the presence of RanGTP in order to test whether the nuclear import of NM1 follows the canonical nuclear import pathway.

Complexes containing Str-GFP-NM1-(Q123.T) and associated importins were purified from electroporated HEK293T cells using streptactin affinity column and incubated with recombinant Q69L mutant of Ran, preloaded with GTP. This mutant is not able to hydrolyze GTP [28] and should cause elution of importins from the Str-GFP-NM1-(Q123.T) column The activity of Q69L mutant of Ran was confirmed by its ability to dissociate importin β1 from its well known cargo, SV40 NLS (Fig. 5E). In contrast to SV40 NLS, the GFP-NM1-(Q123.T) remained associated with importin 5 even in the presence of RanGTP Q69L. As shown by western blot (Fig. 5E), the complex of GFP-NM1-(Q123.T) and importin 5 co-eluted from the column by the addition of biotin that disrupts the binding of Str-GFP-NM1-(Q123.T) to the streptactin resin. Taken together, these data suggested that the NM1 nuclear import does not follow the canonical nuclear import pathway regulated by GTPase Ran.

### Overexpression of calmodulin negatively influences NM1 nuclear import

**Figure 6:**
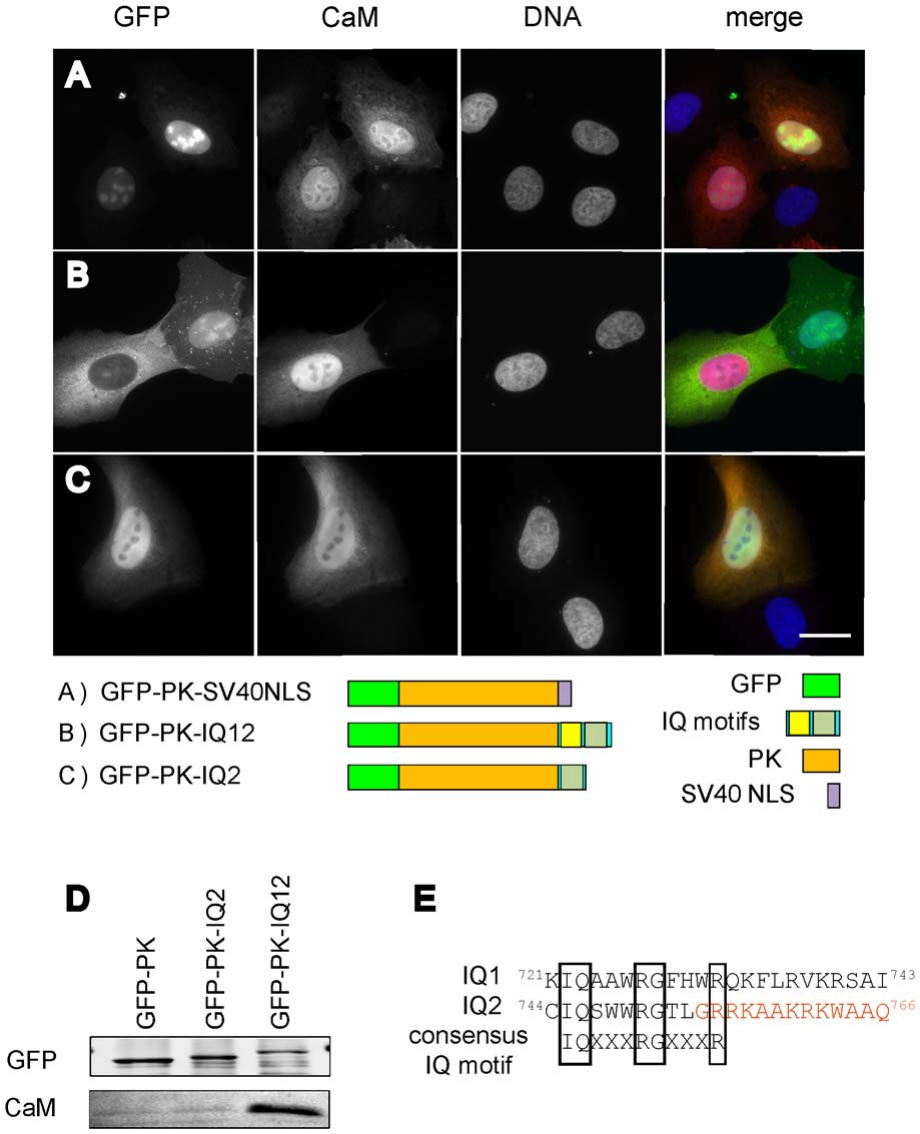
Overexpression of calmodulin influences the nuclear import of NM1. U2OS cells were co-transfected with GFP-PK constructs containing IQ domains, and calmodulin. Calmodulin was visualized using specific antibody (**A**,**B**,**C**). Scale bar 10 µm. HEK293T cells electroporated with the same constructs as in (**A**,**B**,**C)**. Whole cell extracts were subjected to immunoprecipitation with anti-GFP nanobody. Bound proteins were resolved on SDS-PAGE and transferred to nitrocelulose. GFP and CaM were visualized using specific antibodies (**D**). (**E**) Comparison of IQ1 and IQ2 sequences. The consensus IQ motif is shown below. The NM1 NLS sequence is highlighted in red.

Neck region of NM1 is characterized by the presence of IQ motifs that bind calmodulin in Ca^2+^-dependent manner [29]. As NLS sequence of NM1 is present within one of these IQ motifs, we tested the influence of increased calmodulin levels on the NM1 localization. When GFP-PK-IQ12 was co-expressed with calmodulin in U2OS cells, elevated levels of calmodulin blocked the nuclear import of the IQ12 construct (Fig. 6B). Calmodulin, on the other hand, did not block the import of GFP-PK-IQ2 (Fig. 6C). Importantly, calmodulin did not inhibit the nuclear import of the GFP-PK-SV40 NLS construct, suggesting that the observed effect is not a general inhibition of nuclear import pathways (Fig. 6A). To compare the amount of calmodulin associated with GFP-PK-IQ2 and GFP-PK-IQ12 we immunoprecipitated the proteins from extracts of electroporated HEK293T cells using GFP-trap magnetic beads. As shown in Fig. 6D calmodulin associated with GFP-PK construct only when both IQ domains were present (GFP-PK-IQ12). In conclusion calmodulin binding to IQ12 appears to regulate nuclear import of NM1.

## Discussion

Nuclear myosin 1 is ubiquitously expressed protein that localizes to the nuclei of all cell types tested so far with the exception of cells in germinal stage of spermiogenesis [3]. Our previous work described the dynamics of intranuclear relocalization of NM1 [8], [19] and involvement of NM1 in important nuclear processes – namely gene transcription. In this paper, we further contribute to the knowledge of NM1 cellular trafficking by describing the dynamics of its nuclear import and identification of the sequence that is necessary for the nuclear entry of NM1.

### NM1 contains NLS within the IQ domain

We used tagged constructs in search for the NLS of NM1. By deletions and truncations of the full length protein, we narrowed down the region of NM1 required for its nuclear import to a short sequence within the second IQ motif of the neck domain. The sequence contains clusters of basic amino acids intermingled with non-polar amino acids and mutation of the basic residues into alanines blocked the nuclear import of NM1. The NLS does not resemble to any of the NLSs already described in the literature and, thus, it might be expected to have some unique properties. Similarly to NM1, also the neck of other unconventional myosin, myosin Vb, contains IQ sequence, that was shown to be responsible for nuclear and nucleolar localization of this myosin. Furthermore, it also mediates interaction with RNA pol I [30]. An IQ motif of another actin and PIP2 binding protein, the neural Wiskot-Aldrich syndrome protein (N-WASP), serves as an NLS [31]. In conclusion, the ability to drive nuclear import appears to be common to various IQ motifs.

### Role of IQ2 in plasma membrane localization of NM1/Myo1c

NM1/Myo1c neck-tail domain (NM1-(Q123.T)), was shown previously to associate with the plasma membrane through interaction with PIP2. This interaction was assigned to the putative PIP2-specific PH domain in the tail region of myo1c [20] and/or another less specific to the neck region [32]. Interestingly, NM1 mutants which either lacked the second IQ motif or the basic residues within the second IQ sequence were misslocalized from the plasma membrane to the cytosol (Fig. 3B and 3C). These data are in agreement with previously published work that identified the IQ2 as additional plasma membrane binding site [32].

### Mechanism of NM1 nuclear import

The basic mechanism of NM1 nuclear import appears to involve karyopherins, because importin 5, importin 7 and importin-β1 were found to be associated with both overexpressed and endogenous NM1. The interaction of importin 5 with NM1 NLS seems to be specific since NM1 mutant lacking the basic amino acids in NLS did not bind this karyopherin.

Canonical importin-mediated nuclear entry is controlled by the nuclear RanGTP which, upon binding to importins, releases their cargos. Surprisingly, the complex of NM1 with importin 5 is stable in the presence of RanGTP (Fig. 5E) and its nuclear import is rather dependent on the levels of calmodulin (Fig. 6B). This suggests that nuclear import of NM1 is mediated by a non-canonical pathway. Indeed, such a calmodulin-dependent and Ran-independent nuclear import pathway has been shown to regulate the nucleocytoplasmic localization of several transcription factors (SOX9, SRY, c-Rel). The N-terminal domain of SRY and SOX9 contains a calmodulin-binding domain followed by an NLS [33]. It was shown that calmodulin binding stimulates the nuclear entry of SRY and SOX9 [34], [35]. On the other hand the NF-*κ*B/Rel family protein c-Rel binds Ca^2+^-calmodulin via sequence near the NLS and this binding blocks its nuclear accumulation [36]. The NLS of NM1 resides in close vicinity of the second IQ motif. We showed that IQ2 alone is able to drive the translocation of heterologous construct GFP-PK to the nucleus (Fig. 2J). Interestingly, IQ1 seems to play a key role in regulation of NM1 nuclear import (Fig. 6B). In presence of elevated levels of calmodulin, IQ1 mediated the inhibition of nuclear import (Fig. 6B) and it also substantially increased the binding of CaM the IQ2 (Fig. 6D).

On the other hand, the crystallographic [37] and biochemical studies have shown that calmodulin binds the IQ motifs of unconventional myosins in Ca^2+^-free state and that elevated Ca^2+^ dissociates calmodulin from the neck of Myo1c [29]. More recent study showed that in the absence of calcium, Ca^2+^-free calmodulin (apo-CaM) was bound to the IQ1 with highest affinity whereas in the presence of calcium IQ1 dissociated Ca^2+^-CaM most rapidly. Ca^2+^ - induced dissociation of calmodulin molecules from the neck increases ATPase rate and inhibits the motility of Myo1c [38]. We propose a scenario in which the calmodulin occupies the IQ12 at low Ca^2+^ cellular levels preventing the importin from binding to NM1. The Ca^2+^ oscilations which occur during G1 phase of cell cycle [39] or which follow the signal transduction events [40] might cause the calmodulins to dissociate from the NM1 neck. As a result, the CaM-free IQ2 will subsequently bind to the import receptor that transports the NM1 to the nucleus. Taken together, at the cellular level, both the motor function of NM1/Myo1c and its localization appear to be dynamically regulated by transient transient changes in Ca^2+^ concentration. Obviously, further experiments are needed to reveal the details of the mechanism.

### Nuclear localization of NM1/Myo1c

The fact that both Myo1c and NM1 contain the same NLS sequence points to the question whether also Myo1c would be present in the nucleus along with NM1. NM1 was first detected in the nucleus in 1997 [1] using the antibody specific to N-terminus of NM1. Myo1c has never been reported in the nucleus since the antibody directed to the C-terminus [41] does not label the nuclei of cells. Intriguingly, upon transfection with either NM1 or Myo1c, the nuclear signal could be readily detected also by this antibody (Fig. 4B). One plausible explanation of this discrepancy would be that the epitope is probably masked by nuclear binding partners, posttranslational modifications or adopts a different conformation. However, the steady-steate cytosol to nucleus distribution of endogenous NM1 and Myo1c in mouse liver is approximately 70% (cytosol) to 30% (nucleus) Fig. 3E. Therefore another possible intepretation is that the level of endogenous nuclear myosins at steady state is is below the detection limit of the antibody directed to the C-terminus.

In conclusion, our work revealed a novel NLS sequence responsible for nuclear translocation of NM1. This sequence acts as an efficient NLS when fused to different otherwise cytosolic proteins, while the AA 1–16 lacks this capacity. This means that NM1 does not need N-terminal sequence for nuclear import, and that both NM1 and Myo1c can function in the cell nucleus which is supported by detection of both isoforms in the cell nucleus. Finally, our work suggests a complex regulation of myosin 1c nuclear import, mediated by both CaM and importins. This data opens additional interesting questions: Do the two myosins serve the same functions in the nucleus and in the cytoplasm? Do these two myosins have the same molecular properties (e.g. binding to actin, nucleotide, mechanism of strain-dependent release of ADP) or do they somehow differ? Are they tuned to serve the same function at the level of cell, tissue and organism? Obviously, further investigation is needed to answer these questions.

## Materials and Methods

### Antibodies

In immunofluorescence and co-immunoprecipitation experiments we used affinity purified antibody directed to the NM1 N-terminus M3567 (Sigma), antibodies to lamin B (M-20, Santa Cruz Biotechnology), V5-tag (Serotec), V5-tag (V8137-Sigma), FLAG-tag (Stratagene) and to calmodulin (Upstate, cat. No. 05-173). Anti-importin 5 (sc-17802), importin beta (sc-1919) and importin 7 (sc-55235) were purchased from Santa Cruz Biotechnology. R2652 rabbit polyclonal antibody against the tail domain of Myo1c was kindly provided by Peter G. Gillespie, Oregon Hearing Research Center and Vollum Institute [41]; rabbit polyclonal anti-NM1 for western blots was kindly provided by Piergiorgio Percipalle [5], and EGFP antibody was purchased from Invitrogen (cat. No. A11122).

### Cells and transfections

Cell lines cells were obtained from American Type Culture Collection. NIH/3T3 (ATCC No. CRL-1658), U2OS (ATCC No. HTB-96), HeLa (ATCC No. CCL-2) and HEK 293T/17 (ATCC No CRL-11268) were kept in DMEM supplemented with 10% fetal bovine serum (FBS) in 5% CO2/air, 37°C, in humidified atmosphere. HeLa S3 (ATCC No. CCL-2.2) were kept in S-MEM supplemented with 5% FBS and grown in spinner flasks. The U2OS cells were transfected with FUGENE 6 (Roche) according to the manufacturer's protocol, fixed after 48 h and either observed directly under the microscope or labeled with antibodies. HEK293T cells were electroporated using GenePulser (Biorad) electroporator as described [42]. The efficiency of electroporation was about 90%.

### Immunofluorescence microscopy

Cells grown on coverslips were fixed with freshly prepared 3% formaldehyde for 10 minutes, permeabilized with 0.1% Triton X-100 in PBS for 10 minutes, incubated with primary antibodies for 1 hour at room temperature. Primary antibodies were diluted in PBS containing 0.05% Tween-20 (PBST) to 16 µg/ml (NM1), 5 µg/ml (lamin B1), 5 µg/ml (calmodulin), 1 µg/ml (V5-tag), 5 µg/ml (Flag-tag). After washing in PBST, coverslips were incubated with FITC or Cy5-conjugated goat anti-rabbit or goat anti-mouse secondary antibodies (Jackson ImmunoResearch). Coverslips were mounted with Mowiol (Sigma) containing DABCO (Sigma) as an anti-fading agent and 0.1 µg/ml DAPI (Sigma), and observed under fluorescent or confocal microcopes (LEICA DM 6000, LEICA DMI 6000, LEICA TCS SP5 AOBS TANDEM). Brightness and contrast of captured digital images was adjusted with Photoshop software (Adobe).

### Cell synchronization

U2OS cells were treated with nocodazole (80 ng/ml or 400 ng/ml) for 16 h. Mitotic cells were washed off the dish with medium, spun down and resuspended in fresh medium, and seeded on coverslips. Cells on coverslips were cultivated further in fresh medium and fixed 2,4,6,8, and 10 hours after the nocodazole block. Aphidicolin (1 µg/ml) was applied for 16 hours, cells were washed, cultivated in fresh medium, and then fixed 2, 7, 11, 17, and 22 hours post aphidicolin block. NIH 3T3 cells were synchronized by mitotic shake-off. Harvested cells were seeded on poly-lysine coated coverslips, and allowed to attach for 15 min. HeLa cells, used for co-immunoprecipitation of endogenous NM1, were incubated for 16 hours with nocodazole (400 ng/ml), washed in PBS, and then cultivated in complete medium for additional 3 h prior to the harvest.

### Plasmid DNA preparation

NM1-GFP, GFP-NM1, Myo1c-GFP, NM1-V5, Myo1c-V5 were obtained by ligation of full length mouse NM1 (amino acids 1–1024) and Myo1c (aa 1–1028) [2] cDNA into pEGFP-C3, pEGFP-N3 (Clontech) and pcDNA3.1/V5-His (Invitrogen) vectors. Truncations containing head (H) neck with IQ domains (Q123) and tail (T) domains were generated using inverse PCR. NM1-V5-(H) (aa 1 to 716) was generated from NM1-V5.GFP-NM1-(Q123.T) (aa 712 to 1044),GFP-NM1-(Q3.T) (aa 763 to 1044), GFP-NM1-(Q123.T -Δ853) (aa 712 to 853), GFP-NM1-(Q12) (aa 712 to 770) were constructed from GFP-NM1 using standard cloning methods. For inspection of NLS-peptide localization, we produced a testing construct GFP-PK that contains in-frame fusion of EGFP and cytosolic enzyme pyruvate kinase (PK) [22], GFP-PK-IQ-12 was produced by ligation NM1-(Q12) sequence into GFP-PK vector. GFP-PK-IQ1 (aa 712 to 740), GFP-PK-IQ2 (aa 739 to 766) GFP-PK-NLS ^(NM1)^ (aa 754 to 766) were generated by PCR deletions from GFP-PK-IQ1,2. Nt-GFP-PK was produced by ligation of NM1 N-terminal sequence (aa 1–16) in front of EGFP in GFP-PK vector. Ligation of the OneStrep tag sequence (IBA) in front of EGFP in the pEGFP-C3 vector generated Str-GFP. Str-GFP-NM1-(Q123.T) was produced by ligating the Q123.T (aa 712 to 1044) sequence into Str-GFP vector. Calmodulin cDNA was prepared from HeLa total cell RNA using RT-PCR and cloned into pcDNA3.1 vector (Invitrogen). Bacterial expression vector pET-Str-His was generated by ligation of OneStrep sequence into the pET28b vector (Novagen). Str-IQ12-His was produced by an in-frame ligation of the PCR-amplified fragment of NM1-(Q12) (aa 712 to 770) between the OneStrep- and His-tag. Point mutations in the NLS sequence of NM1 were generated by the site directed mutagenesis protocol (Stratagene). Bacterial expression plasmid pQE-RanQ69L was kindly provided by Prof. Dirk Görlich. Detailed description of all cloning procedures is available upon request. Recombinant proteins were expressed in bacteria and purified using Ni-NTA agarose column as described [28], [38].

### Pull-down assays and immunoprecipitation

Digitonin extract from suspension HeLa cells, prepared as described [43], was diluted to 2 mg/ml of total protein in lysis buffer (50 mM HEPES pH 7.4, 150 mM NaCl, 75 mM potassium acetate, 5 mM magnesium acetate, 1 mM DTT, protease inhibitors). After dilution, purified bacterially expressed Str-IQ12-His was added to the lysate. After 3 hours of incubation, the extract was centrifuged to remove precipitated proteins and supernatant was further incubated for 1 hour with StrepTactin Beads (IBA) to capture the bait and associated proteins. Beads were briefly washed 3 times with 1 ml of the lysis buffer followed by brief wash with IBA wash buffer (100 mM Tris pH 7.5, 100 mM NaCl, 1 mM EDTA) and captured proteins were eluted from beads with the wash buffer supplemented with 2 mM biotin. HEK 293T cells electroporated with Str-GFP-NM1-(Q123.T) or Str-GFP were collected by trypsinization into serum-containing medium. After centrifugation 300 g/3 minutes, the cells were washed twice with ice-cold PBS and extracted twice with lysis buffer containing 50 mM HEPES pH 7.4, 150 mM potassium acetate, 5 mM magnesium acetate, 1 mM DTT, 1 mg/ml digitonin (Fluka), EDTA-free COMPLETE inhibitors (Roche). After 4 hours of incubation with StrepTactin resin, the captured protein complexes were washed briefly 3 times with 1 ml of the lysis buffer followed by wash with 1 ml of IBA wash buffer. Proteins were eluted from beads with 2 mM biotin added into the wash buffer. The experiments with RanQ69L mutant were performed as described above, with the exception that after the 3^rd^ wash a half of the beads was incubated for 10 min with buffer containing recombinant RanQ69L and the other half was incubated only in buffer. Elution with Ran mutant was repeated twice and remaining proteins were eluted from beads with IBA elution buffer containing 2 mM biotin. Eluates were concentrated ultrafiltration (Ultracel 10K, Milipore) and resolved on 6–20% gradient polyacrylamide gel.

Endogenous NM1 was immunoprecipitated from adherent HeLa cells synchronized with nocodazole. Cells were extracted twice in lysis buffer (50 mM HEPES pH 7.4, 150 mM NaCl, 75 mM potassium acetate, 5 mM magnesium acetate, 1 mM DTT, 1 mg/ml of digitonin and protease inhibitors), lysates were clarified by centrifugation (10 min, 16 000 g, 4°C), and incubated with beads containing either covalently bound antibody antibody to NM1 (Sigma, cat no M3567) or to EGFP (Exbio, Czech Republic, cat no 11-473-C100). After 3 washes in 1 ml of lysis buffer beads were washed in 1 ml of 50 mM amonium bicarbonate pH 7.5 to remove salts and detergent. Bound proteins were eluted twice with 500 µl of 500 mM amonium hydroxide. Eluates were evaporated using SpeedVac concentrator (Savant, Holbrook, NY, USA), dry pellets were resuspended in 20 ul of 1× SDS loading buffer, boiled and resolved on 6–20% gradient SDS PAGE. After transfer to nitrocelulose proteins were visualized using specific antibodies.

GFP-PK constructs in Fig. 6D were immnuoprecipitated from lysates of electroporated HEK23T cells as follows. Cells were harvested by trypsinization, washed in PBS and lysed in lysis buffer (150 mM NaCl, 50 mM Tris-HCL pH-7.5, 10 mM EGTA, 2 mM EDTA, 1% Triton X-100, protease inhibitors ROCHE). After clarification by centrifugation (10 min, 16 000 g, 4°C), supernatans were incubated with 20 µl of GFP-trap magnetic particles (ChromoTek GmbH, Germany). After 5 washes in 1 ml of lysis buffer particles were washed in 1 ml of 50 mM amonium bicarbonate pH 7.5 to remove salts and detergent. Bound proteins were eluted twice with 500 µl of 500 mM amonium hydroxide. Eluates were evaporated using SpeedVac concentrator (Savant, Holbrook, NY, USA), dry pellets were resuspended in 20 ul of 1× SDS loading buffer, boiled and resolved on 6–20% gradient SDS PAGE. After transfer to nitrocelulose proteins were visualized using specific antibodies.

### Proteolytic digestion and sample preparation

Protein bands were cut from the gel, sliced into the small pieces, and decolorized in sonic bath at 60°C several times with 0.1 M 4-ethylmorpholine acetate (pH 8.1) in 50% acetonitrile (ACN). After complete destaining, proteins were reduced by 50 mM TCEP in 0.1 M 4-ethylmorpholine acetate (pH 8.1) for 5 min at 80°C and alkylated using 50 mM iodoacetamide in 0.1 M 4-ethylmorpholine acetate (pH 8.1) for 30 min in dark at room temperature. Then, the gel was washed with water, shrunk by dehydration with ACN and reswollen in water. The rehydratation and dehydration of the gel was repeated twice. Next, the gel was reswollen in 0.05 M 4-ethylmorpholine acetate (pH 8.1) in 50% acetonitrile (ACN) and then the gel was partly dried using a SpeedVac concentrator (Savant, Holbrook, NY, USA). Finally, the gel was reconstituted with cleavage buffer containing 0.01% 2-mercaptoethanol, 0.05 M 4-ethylmorpholine acetate (pH 8.1), 10% ACN, and sequencing grade trypsin (Promega, 10 ng/µl). Digestion was carried out overnight at 37°C; the resulting peptides were extracted with 30% ACN/0.1% TFA and subjected to mass spectrometric analysis.

### Mass spectrometric analysis

Mass spectra were acquired in the positive ion mode on a MALDI-FTMS APEX-Ultra (Bruker Daltonics, Bremen, Germany) equipped with 9.4 T superconducting magnet and SmartBeam laser. The acquisition mass range was 700–3500 m/z and 512k data points were collected. A 280 V potential was applied on the MALDI plate. The cell was opened for 2500 ms, 4 experiments were collected for one spectrum where one experiment corresponds to 300 laser shots. The instrument was externally calibrated using PepMix II peptide standard (Bruker Daltonics, Bremen, Germany). It results in typical mass accuracy below 2 ppm. A saturated solution of α-cyano-4-hydroxy-cinnamic acid in 50% ACN/0.2% TFA was used as a MALDI matrix. A 1 µl of matrix solution was mixed with a 1 µl of the sample on the target and the droplet was allowed to dry at ambient temperature. After the analysis the spectra were apodized using square sin apodization with one zero fill. The interpretation of mass spectra was done using DataAnalysis version 3.4 and BioTools 3.2 software packages (Bruker Daltonics, Billerica, MA). Proteins were identified by peptide mass fingerprinting (PMF) using a search algorithm MASCOT (Matrix Science).

### Generation of the NM1 knock-out mice

To generate NM1-KO mice, loxP-recombination sites were introduced into NM1 gene by homologous recombination in R1 embryonic stem cell line [44]. Cre-mediated recombination in germline cells, achieved by cross breeding with the meu-cre expressing mice [45] resulted in removal of the loxP-flanked exon-1 from the mouse NM1 genomic sequence (sequence from −165 to +116 base pairs from NM1 translation initiation site). In the mutant NM1 allele, only the start codon initiating the translation of Myo1c is present. As a result, only Myo1c protein is expressed in all tissues. Mice were genotyped using genomic PCR, and the absence of NM1 protein was confirmed by Western blotting (Venit et al., in preparation).

### Isolation of nuclei from mouse liver

Nuclei from mouse liver were isolated as described [46]. Briefly, mice were killed by CO_2_ and liver was homogenized in ice-cold buffer A (250 mM sucrose, 5 mM MgCl_2_, 10 mM HEPES pH 8) in glass Dounce homogenizer. The homogenate was spun down (600 g/10 min), the supernatant was taken as the cytosolic fraction and the pellet was washed once in buffer A. The crude nuclear pellet was resuspended in buffer B (2.0 M sucrose, 1.5 mM MgCl_2_, 10 mM HEPES pH 8) and centrifuged 30 minutes/16000 g. Purified nuclei were resuspended in buffer Z (62.5 mM Tris pH 6.8, 10% glycerol, 2% SDS), heated to 90°C for 10 minutes, sonicated, and centrifuged again (16000 g/10 min). The amount of protein in the supernatant was measured using BCA (Pierce).
